# Case Report: Unexpected distal airway obstruction in prematurity: a fatal case of intratracheal decidual aspiration and a call for revisiting resuscitation algorithms

**DOI:** 10.3389/fped.2026.1840549

**Published:** 2026-05-29

**Authors:** Haoqiang Xie, Jianbo Li, Jinfeng Li, Jinfeng Liao, Bang Du, Ning Li, Xiaoguang He

**Affiliations:** Department of Neonatology, Dongguan Children’s Hospital Affiliated to Guangdong Medical University, Dongguan, China

**Keywords:** distal airway obstruction, neonatal resuscitation, decidua, aspiration, preterm infant, case report, algorithm pitfall, False-Patency sign

## Abstract

**Background:**

Unexpected distal airway obstruction is a critical challenge during neonatal resuscitation. Aspiration of intrauterine tissue is an uncommon cause that may not respond to standard algorithms. Through comparative analysis with three previously documented cases, this report aims to clarify the clinical features, diagnostic pitfalls, and key outcomes for this entity, emphasizing the need for heightened clinical awareness and technological advances.

**Case presentation:**

A male infant was born at 30⁺⁵ weeks via vaginal breech delivery due to spontaneous preterm labor. The mother completed antenatal corticosteroids for lung maturation 4 days prior. The infant exhibited apnea and bradycardia. Standard neonatal resuscitation failed. A DOPE assessment revealed no correctable problems; a suction catheter passed easily, creating a deceptive “False-Patency” sign. Deeper suctioning yielded only scant secretions. Chest compressions began at 18 min after birth. Simultaneously, intravenous epinephrine (1:10,000) was administered every three minutes, and a resuscitation bag delivered positive-pressure ventilation. These measures continued for 42 min (until one hour of life). Approximately 60 min after delivery, with chest compressions and ventilation still in progress, two bean-sized tissue fragments were pushed out of the trachea. After removal via endotracheal tube exchange, the infant achieved return of spontaneous circulation (ROSC). Within two minutes, heart rate rose to 140 beats/minute, and SpO₂ reached 93%. Chest compressions were then halted. Histopathology confirmed decidual tissue. Despite maximal support, the infant died from irreversible multi-organ failure.

**Conclusion:**

This case illustrates that aspiration of intrauterine decidual tissue is a rare but devastating cause of unexpected distal airway obstruction in preterm infants. Findings from all four reported cases indicate that standard algorithms (MRSOPA/DOPE) are ineffective. The “False-Patency” sign—easy passage of a suction catheter despite absent chest rise and breath sounds—is a major diagnostic trap, delaying identification of a solid, peripherally impacted foreign body. A strong suspicion for this condition is critical. While specialized airway visualization instruments for preterm neonates remain a long-term goal, the primary solution lies in cognitive vigilance—specifically, recognizing this unusual condition and its misleading “False-Patency” indicator.

## 1 Introduction

The occurrence of an unexpected distal airway obstruction in a neonate represents a life-threatening situation during resuscitation efforts. We define “distal airway obstruction” as ventilatory failure resulting from a solid or semi-solid foreign body lodged distal to the endotracheal tube tip—typically within a main or lobar bronchus—as opposed to obstructions at the glottic or subglottic level or within the tube itself. When such an airway is “unexpected"—arising in the absence of identifiable antenatal risk factors—it presents distinctive diagnostic and management difficulties (1). Although standardized neonatal resuscitation algorithms, including MRSOPA (Mask, Reposition, Suction, Open mouth, Pressure, Airway) and DOPE (Displaced, Obstructed, Pneumothorax, Equipment), serve as fundamental components of clinical practice (2), they may be insufficient for addressing airway obstruction stemming from uncommon causes, such as aspiration of intrauterine tissue (3–5).

Currently, the English-language medical literature contains only three documented instances of neonatal airway obstruction caused by aspirated placental or decidual material (3–5). Among these reported cases, the single fatal outcome—described by van Wylick and colleagues (5)—demonstrates remarkable parallels with the case we report in this manuscript. This resemblance affords a distinctive chance to advance beyond a standard case presentation. In this article, we describe a new fatal instance of an unexpected distal airway obstruction in a preterm infant resulting from intratracheal aspiration of decidual tissue. Through conducting a comprehensive comparative evaluation of the previously documented cases—with specific focus on the fatal case (5)—this manuscript endeavors to: (1) synthesize the clinical phenotype characterizing this uncommon condition; (2) recognize shared diagnostic and therapeutic obstacles that cause standard algorithms to fail; and (3) obtain evidence-based recommendations to inform clinical management and direct future investigations.

## 2 Case presentation

A male infant was born at 30⁺⁵ weeks of gestation following a vaginal breech delivery. The preterm birth occurred spontaneously, which was the reason for delivery. His mother, a 29-year-old woman (G3P1), was hospitalized due to threatened preterm labor. She received a complete course of antenatal corticosteroids 4 days before delivery to promote fetal lung maturation, as well as magnesium sulfate for neuroprotection of the fetus. Intrapartum fetal monitoring revealed transient bradycardia during uterine contractions, although variability and accelerations remained normal between contractions. The amniotic fluid was clear, and the infant's birth weight was 2 kg.

Upon delivery, the neonate presented with apnea, pallor, hypotonia, and bradycardia (heart rate 70 beats per minute). The Apgar scores were 1, 1, and 1 at 1, 5, and 10 min, respectively. Initial stabilization measures—including thermal care, proper positioning, oropharyngeal suctioning, and tactile stimulation—yielded no clinical improvement. Positive-pressure ventilation was subsequently initiated using a T-piece resuscitator (initial parameters: FiO₂ 30%, PEEP 5 cm H₂O, PIP 20 cm H₂O); however, this failed to generate adequate chest expansion or elevate the heart rate. The MRSOPA algorithm was then implemented in a stepwise fashion, comprising mask reseating, repositioning, suctioning (which produced thin, white mucus), ensuring an open airway, increasing PIP to 30 cm H₂O, and titrating FiO₂ to 100%. Given the persistence of ineffective ventilation, endotracheal intubation was performed at 1 minute and 30 seconds of life using a 3.0 mm endotracheal tube.

**Figure 1:**
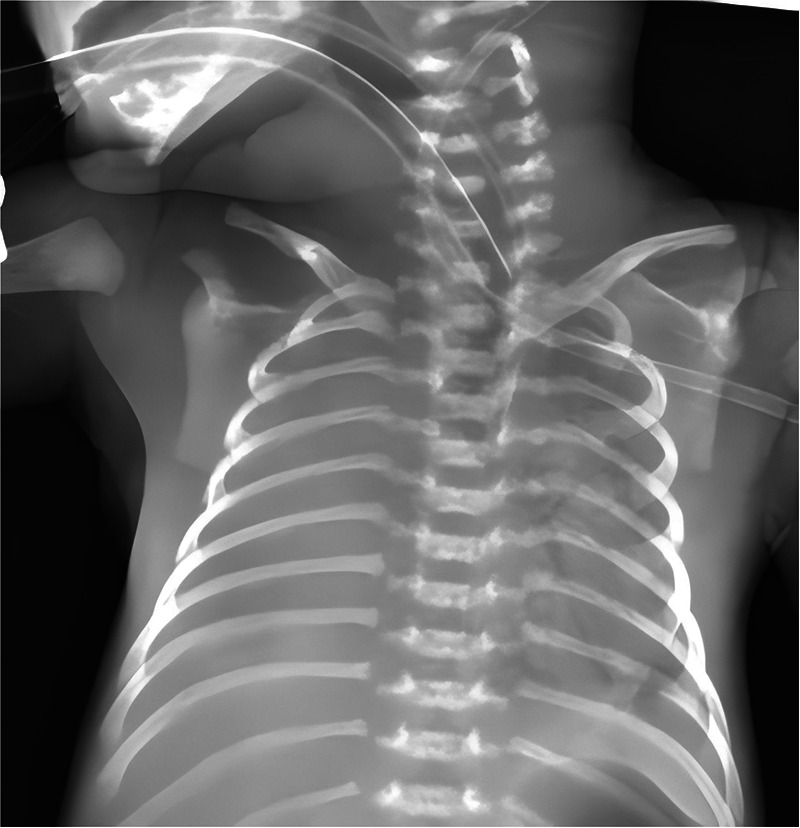
Bedside chest x-ray during resuscitation showing neonatal respiratory distress syndrome with diffuse ground-glass opacification and air bronchograms, more pronounced on the right.

After intubation, bilateral chest wall movement and breath sounds remained undetectable. A systematic evaluation employing the DOPE checklist was performed. Tube displacement was ruled out by re-visualizing the tube passing through the vocal cords via laryngoscopy, confirming the depth of 8 cm at the lip. Pneumothorax was ruled out by negative transillumination and, most definitively, by immediate bedside point-of-care ultrasound showing a “lung pulse” and no lung point. Equipment malfunction was excluded by checking circuit integrity. Obstruction was initially considered less likely because a standard 6Fr suction catheter passed easily without resistance down the endotracheal tube. Arterial blood gas analysis indicated severe mixed acidosis (pH 7.042, pO₂ < 10 mm Hg, pCO₂ 78 mm Hg, lactate 5.71 mmol/L). Further interventions included exchanging the endotracheal tube (replacing the 3.0 mm with a 3.5 mm), elevating PIP to 40 cm H₂O, and conducting deeper suctioning maneuvers. Deeper suctioning was then attempted. On multiple attempts, a 6Fr suction catheter passed easily and without resistance through the endotracheal tube, but further advancement met resistance, yielding only a small amount of thin, white secretions. Bedside lung ultrasound combined with chest radiography (Figure 1) excluded pneumothorax and diaphragmatic hernia; imaging findings aligned with neonatal respiratory distress syndrome, revealing right lung consolidation accompanied by a lung pulse sign.

For this infant experiencing refractory ventilatory failure during active resuscitation, the differential diagnosis included severe neonatal respiratory distress syndrome (NRDS), tension pneumothorax, and critical airway obstruction caused by either a foreign body or a structural anomaly. Severe NRDS was initially regarded as the most likely possibility. However, the immediate and complete failure of ventilation noted after successful endotracheal intubation was atypical of uncomplicated RDS. Therefore, congenital tracheal stenosis or distal airway obstruction from a foreign body was raised as a consideration, though neither could be promptly confirmed.

Chest compressions were initiated at 18 min after delivery. Simultaneously, the infant received intravenous epinephrine (1:10,000) every three minutes, and positive-pressure ventilation was delivered through a resuscitation bag. This combined resuscitation protocol was sustained continuously for 42 min, lasting until one hour of life. Despite these intensive efforts, oxygen saturation stayed within the range of 10%–20%. An 8Fr catheter was used for deep suctioning, yet no aspirate of abnormal material was obtained. Around 60 min after delivery, while chest compressions and positive-pressure ventilation were still in progress, two tissue fragments, each bean-sized and measuring approximately 5 × 3 mm, were pushed out from the trachea and became stuck at the endotracheal tube immediately beyond the incisor line. Since these fragments caused complete tube obstruction and could not be withdrawn by suction, the endotracheal tube was replaced with a new one. Following removal of the fragments through this tube exchange, the infant attained return of spontaneous circulation (ROSC). Within two minutes, the heart rate climbed to 140 beats per minute, and SpO₂ reached 93%. Chest compressions were discontinued as soon as ROSC was confirmed, and the lung pulse sign disappeared on ultrasound examination. Surfactant (120 mg) was delivered, and the infant was transported to the neonatal intensive care unit while receiving conventional mechanical ventilation. Subsequent inspection of the umbilical cord and placenta revealed no macroscopic abnormalities, with no observed disruptions or defects.

**Physical Examination on Admission (at 1 h and 8 min of age):** Following transfer to the neonatal intensive care unit, the infant presented in a comatose state, characterized by absent pupillary light reflexes and generalized hypertonia. Assessment of vital signs demonstrated hypothermia (35.8 °C), tachycardia (145 beats per minute), ventilator-dependent respiratory rate of 50 breaths per minute, and a mean blood pressure of 33 mm Hg.

**Figure 2:**
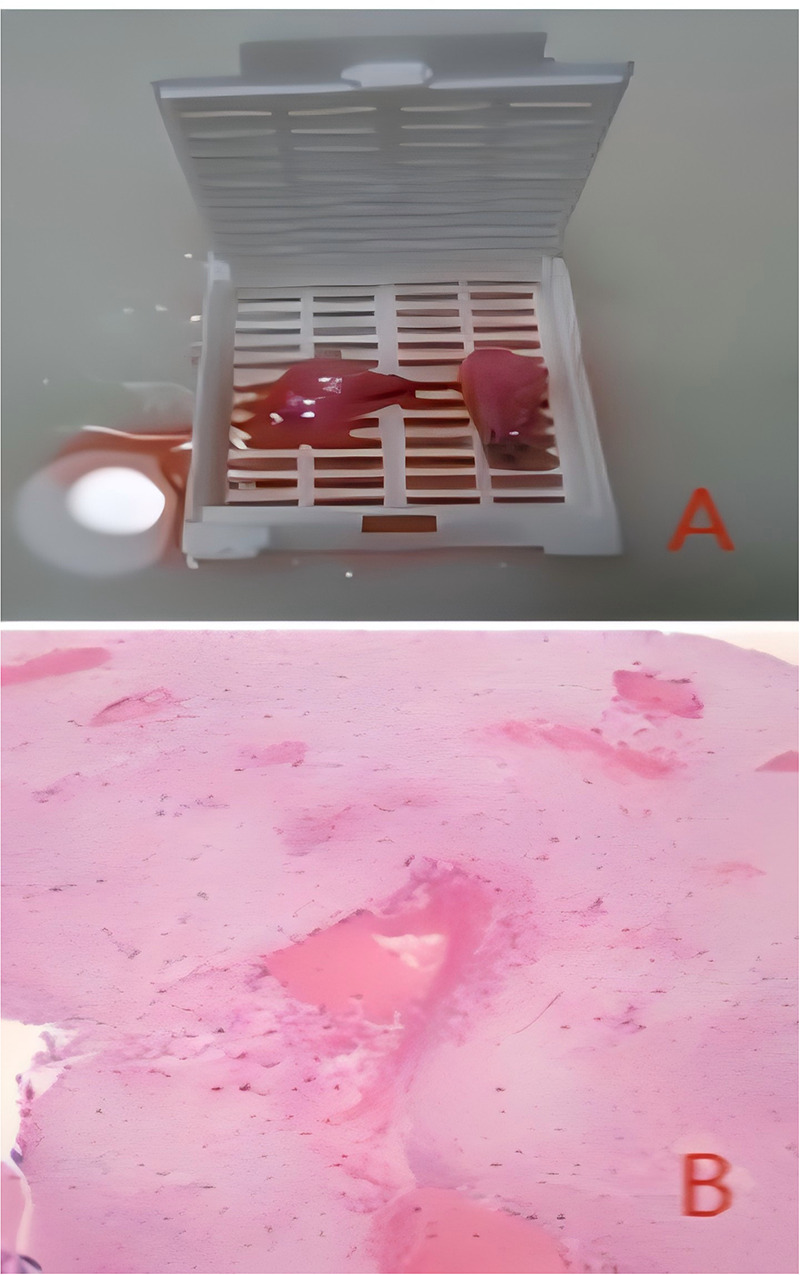
**(A)** tissue fragments suctioned from the trachea during resuscitation, measuring approximately 5 × 3 mm each. **(B)** Hematoxylin and eosin staining (  ×  200 magnification) of the tissue confirmed decidual origin, characterized by sheets of polygonal decidual cells with abundant eosinophilic cytoplasm, surrounded by fibrinoid material.

**Laboratory Findings:** Initial laboratory evaluation revealed profound leukopenia, with the white blood cell count decreasing from 7.49 × 10⁹/L at birth to 0.43 × 10⁹/L by 4 h postpartum. Substantially elevated inflammatory markers were detected, including procalcitonin measuring 70.38 ng/mL and interleukin-6 exceeding 5,000 pg/mL. Biochemical evidence confirmed multi-organ dysfunction, with creatinine 132 μmol/L, urinary microalbumin 864 mg/dL, AST 892 U/L, and ALT 421 U/L. Serial blood gas analyses demonstrated progressive severe acidosis accompanied by hyperlactatemia, reaching a nadir of pH 6.523 with lactate 18.36 mmol/L at 1 h of life. Coagulation profiles showed prolonged prothrombin time (28.4 s) and activated partial thromboplastin time (>120 s), reduced fibrinogen (0.8 g/L), and markedly increased D-dimer (>20 μg/mL), findings consistent with disseminated intravascular coagulation. Subsequent microbiological analysis of sputum and gastric fluid cultures yielded extended-spectrum beta-lactamase–producing Escherichia coli. Histopathological assessment of the tracheal aspirate confirmed the presence of decidual tissue, exhibiting sheets of polygonal cells containing abundant eosinophilic cytoplasm with characteristic fibrinoid deposition (Figure 2).

**Hospital Course (see timeline in Table 1):** The infant's hospital course was complicated by progressive multisystem failure, including severe hypoxic-ischemic encephalopathy, persistent pulmonary hypertension (estimated pulmonary artery pressure 44 mm Hg by echocardiography), acute kidney injury, disseminated intravascular coagulation, and agranulocytosis. Despite maximal supportive care—comprising high-frequency oscillatory ventilation, inhaled nitric oxide, vasopressor support, and broad-spectrum antibiotics—his clinical status deteriorated inexorably. The infant died of irreversible multiple organ failure.

**Table 1:** Summary of neonatal cases with airway obstruction due to aspiration of placental or decidual tissue.

Case	Patient characteristics	Delivery & circumstances	Clinical presentation	Diagnostic method	Management	Outcome	Key observation/pitfall
1 (3)	Preterm, 32 weeks’ gestation	Cesarean delivery; complicated by difficult head extraction.	Immediate tracheal obstruction post-delivery. Initial improvement after intubation was followed by self-extubation and apnea, with no chest wall movement during IPPV.	Direct laryngoscopy visualized tissue obstructing the ETT; histopathology confirmed placental origin.	Endotracheal intubation. A tracheal cast of placental tissue was suctioned under direct laryngoscopy, followed by successful re-intubation.	Normal neurodevelopment at follow-up.	Visualization was key to rapid resolution. Obstruction relieved within minutes.
2 (4)	Male term infant, 38 weeks, 3,140 g	Cesarean delivery indicated for uterine scar and leiomyoma; an extremely difficult procedure.	Low 1-minute Apgar score and respiratory distress. Chest radiograph revealed an opaque hemithorax with mediastinal shift and pneumothorax.	A tissue fragment was retrieved via suction catheter through the ETT; histopathology confirmed placental tissue.	Orotracheal intubation and initiation of high-frequency oscillatory ventilation. The obstructing tissue fragment was suctioned via the ETT.	Rapid clinical improvement with immediate re-aeration of the left lung.	Obstruction relieved relatively quickly via suction. Survived with good outcome.
3 (5)	Preterm female, 29 weeks, 1,435 g	Vaginal breech delivery following preterm labor and antepartum hemorrhage.	Severe perinatal asphyxia (Apgar scores of 1 at 1 and 5 min). Multiple intubation attempts yielded no air entry. Unresponsive to high PIP, epinephrine, and needle thoracocentesis. A suction catheter passed easily.	Postmortem examination identified a decidual tissue fragment (1.2 × 0.2 cm) lodged in the trachea/larynx.	Repeated intubation attempts, administration of high PIP and IV/ET epinephrine, and needle decompression. Resuscitation was discontinued at 27 min.	Death (unresolved airway obstruction).	Suction catheter passed easily, falsely suggesting patency (“False-Patency”). Prolonged, unrecognized complete obstruction leading to death.
4 (Present Case)	Preterm male, 30⁺⁵ weeks, 2,000g	Breech delivery.	Refractory apnea, bradycardia, and severe metabolic acidosis unresponsive to resuscitation. Multiple suction attempts with easy catheter passage.	Tissue fragments were suctioned from the ETT during ongoing resuscitation; histopathological examination confirmed decidual tissue.	Prolonged advanced resuscitation and maximal organ support therapy in the NICU.	Death secondary to multiple organ dysfunction syndrome (MODS).	Suction catheter passed easily (“False-Patency”). Obstruction was not recognized until severe hypoxic-ischemic injury had already occurred, despite adherence to standard protocols.

ETT, Endotracheal tube; HFOV, high-frequency oscillatory ventilation; IPPV, intermittent positive pressure ventilation; MODS, multiple organ dysfunction syndrome; PIP, Peak Inspiratory Pressure; IV, Intravenous; ET, Endotracheal.

## 3 Discussion

The present report documents an uncommon and lethal etiology of unexpected distal airway obstruction in a preterm neonate. Airway obstruction resulting from aspirated decidual tissue proved unresponsive to conventional neonatal resuscitation algorithms. To better characterize this clinical entity and extract maximal clinical value, we present a systematic comparison of our patient's presentation and clinical trajectory with the three previously published cases of neonatal airway compromise attributable to aspirated placental or decidual material (Table 2).

**Table 2:** Differentiating refractory NRDS from distal solid airway obstruction in a preterm infant.

Feature	Severe, refractory NRDS	Distal solid airway obstruction (e.g., decidual tissue)
Response to surfactant	Usually some improvement in FiO2/oxygenation index	No improvement
Chest x-ray	Diffuse ground-glass opacification, air bronchograms	May show asymmetric aeration, or potentially a normal x-ray
Suction catheter passage	Passes easily, may return mucus	Passes easily (“False-Patency” sign)
Response to MRSOPA/DOPE	Generally improves with optimization	No improvement
Breath sounds	Symmetrically decreased or coarse	Either asymmetrically or symmetrically decreased or absent

This comparative evaluation yields several important insights extending beyond the individual case descriptions.

### 3.1 Consistent clinical context and proposed mechanism

The clinical circumstances demonstrate remarkable uniformity across all four reported cases (3–5). Each instance involved a complicated delivery, with three specifically associated with breech presentation or difficult extraction. We hypothesize that this consistent pattern strongly implicates the mechanical forces generated during parturition—particularly with abnormal fetal positioning—as a critical factor promoting the aspiration of placental or decidual fragments. One plausible mechanism involves uterine compression during fetal extraction, forcibly propelling tissue fragments into the oropharynx, followed by their inhalation coincident with the infant's initial breaths. However, we cannot rule out alternative etiologies, such as prenatal aspiration due to in-utero fetal gasping from transient cord compression (suggested by fetal bradycardia), or an underlying chorioamnionitis. Consequently, clinicians should maintain an elevated level of vigilance for atypical airway obstruction when resuscitating infants delivered under such circumstances (6).

### 3.2 The “false-patency” phenomenon: a critical diagnostic pitfall

Most significantly, our analysis identifies a crucial diagnostic trap: the “False-Patency” phenomenon. In both the lethal case described by van Wylick and colleagues (5) and the current case, unimpeded passage of a suction catheter through the endotracheal tube created a deceptive impression of airway patency.

We offer the following more precise definition of the “False-Patency Sign.” During a standard DOPE evaluation, clinicians typically use the ease of suction catheter passage as an initial indicator to exclude obstruction (“O”). The “False-Patency Sign” occurs when the obstructive material is solid or semisolid and is either lodged distally (for example, within a main bronchus) or functions as a ball-valve. Under these circumstances, the suction catheter may advance without resistance alongside the material, or it may reach the distal end of the endotracheal tube without contacting the more peripherally located obstruction, thus creating an illusion of a patent airway (7). As a result, the “O” component of DOPE is not genuinely ruled out but is instead erroneously considered eliminated. A more cautious clinical interpretation is therefore warranted: even when a suction catheter passes with ease, if chest expansion is totally absent and breath sounds cannot be heard, a high level of suspicion for a distal or ball-valve solid foreign body obstruction must be maintained.

A more plausible hypothesis, however, is that the tissue fragment was impacted in a region distal to the tip of the endotracheal tube, beyond the reach of the suction catheter—or even if reached, could not be aspirated out. The positive-pressure ventilation delivered via the resuscitation bag, combined with chest compressions during resuscitation, eventually dislodged this peripheral fragment toward the endotracheal tube, from which it was ultimately cleared by tube exchange. This pathophysiological mechanism, rarely highlighted in earlier publications, clarifies why conventional DOPE assessments—which depend on unobstructed catheter passage to dismiss the possibility of obstruction—can prove dangerously misleading under these circumstances.

### 3.3 Inadequacy of current resuscitation algorithms and anatomical constraints

The limitations of existing resuscitation protocols are vividly demonstrated by these cases. Even when current algorithms prove effective for obstructions that are visible or easily reached (as seen in Cases 1 and 2), their limitations become most obvious in the two lethal cases (Cases 3 and 4) when confronting a more subtle, peripherally situated blockage. The MRSOPA and DOPE protocols were originally devised to address typical causes of ventilation failure, including mask leaks, misplaced tubes, mucus plugs, pneumothorax, and malfunctioning equipment. These protocols are fundamentally ill-equipped to handle a solid, adherent foreign body lodged within the distal airways of a premature newborn (8). The aspirated decidual tissue, being viscous and firm, resists extraction via standard suctioning techniques. Moreover, the inherently small caliber of a preterm infant's airways represents a nearly insurmountable hurdle. For a 30-week preterm neonate, the cricoid cartilage's internal diameter is roughly 3.5–4.0 mm. Contemporary flexible bronchoscopes have outer diameters in the range of 2.2–2.8 mm; even if such a scope can be advanced through a 3.0–3.5 mm endotracheal tube, the remaining space is inadequate for instrument maneuvering or effective suction alongside the scope (9). Additionally, this kind of equipment is seldom accessible in delivery room environments. This circumstance reveals a fundamental shortcoming in contemporary resuscitation guidelines when confronted with this particular, uncommon scenario (10, 11).

### 3.4 Timeliness of intervention determines outcome

Clinical outcome proves decisively dependent on the promptness with which obstruction is recognized and relieved. Comparison across the four cases substantiates this essential observation. In Cases 1 and 2 (3, 4), where obstructing material was extracted relatively expeditiously—within minutes of identification—the infants survived with favorable neurodevelopmental outcomes. In Case 2, the obstructing tissue was successfully retrieved via suction catheter through the endotracheal tube, producing immediate re-aeration. Conversely, in Case 3 (5) and the present case (Case 4), where complete obstruction persisted despite continued resuscitation efforts, mortality ensued. These divergent outcomes emphasize that, notwithstanding maximal compliance with standard protocols, the subtle nature of this obstruction may delay its detection and resolution, ultimately resulting in profound hypoxic-ischemic damage. The extensive multi-organ failure documented in our patient—comprising hypoxic-ischemic encephalopathy, acute kidney injury, disseminated intravascular coagulation, and agranulocytosis—exemplifies the characteristic consequences of severe perinatal asphyxia (12) and compellingly demonstrates the catastrophic repercussions when such obstruction escapes rapid identification.

### 3.5 Potential infectious contribution and other etiologies

The possible role of infection in these cases merits consideration. Both our patient and Case 3 (5) exhibited evidence of infection, with antepartum hemorrhage documented in Case 3 and positive E. coli cultures accompanied by profound leukopenia in our case. The observation of fetal bradycardia accompanied by repeated decelerations during labor raises the possibility of in-utero gasping, which may serve as an alternative route for aspiration occurring before delivery. It remains uncertain whether ascending infection contributes to decidual tissue breakdown or whether the aspirated material itself acts as a focus for bacterial colonization; nevertheless, this link deserves further study.

### 3.6 Implications for clinical practice, multidisciplinary care, and future directions

Collectively, these observations compel advancements on multiple fronts. Taken together, these findings demand progress across multiple areas. The most critical and primary take-home point is cognitive: we advise clinicians to keep a high degree of suspicion for this uncommon diagnosis when encountering the following two features: (1) refractory ventilation failure that fails to improve with MRSOPA/DOPE measures, and (2) the “False-Patency” sign. To assist in differentiating this entity from severe RDS (the most common consideration in the differential diagnosis), we have included a comparison table (Table 3). Second, we recognize the considerable logistical, financial, and training-related challenges associated with introducing specialized airway equipment into delivery rooms. Accordingly, prevention depends on cognitive preparedness and strict protocol adherence. For deliveries that carry high risk (for instance, preterm breech presentation or fetal distress), the delivery team should receive explicit briefing regarding the potential for an unexpected distal airway obstruction and the “False-Patency” sign. Third, management by a multidisciplinary team (MDT) is the optimal approach. Such a team would ideally consist of neonatologists, pediatric anesthesiologists, pediatric otolaryngologists (for possible rigid bronchoscopy), and nursing leadership. However, the urgent nature of these events frequently precludes the timely activation of a formal MDT .Lastly, the long-term aspirational objective remains the development of dedicated, miniaturized, semi-flexible airway visualization tools—for example, ultra-thin bronchoscopes or fiberscopes with outer diameters ≤2.0 mm that retain a working channel adequate for suction and foreign body retrieval—designed specifically for the preterm airway (13). Such instruments could be employed in delivery settings when distal airway obstruction occurs and standard approaches fail. We acknowledge that extensive research and development will be required before such a device becomes clinically practicable. In the meantime, clinical vigilance and prompt recognition of the “False-Patency” sign remain our most potent resources. We further note that antenatal counseling for preterm labor should address the broad range of risks associated with neonatal resuscitation, including the rare chance of an unanticipated airway emergency, while avoiding unnecessary alarm through detailed discussion of highly improbable events such as tissue aspiration.

**Table 3:** Clinical timeline from birth to death.

Time from birth	Actual time	Event/intervention	Key clinical status/findings
0 min	22:29 (Oct 24)	Vaginal breech delivery; Apgar 1,1,1	Apnea, bradycardia (HR 70), pallor
0–10 min	22:29–22:39	PPV, MRSOPA maneuvers, intubation (3.0 mm ETT)	No chest rise, SpO₂ 10%–20%
10–18 min	22:39–22:47	Tube exchange (3.5 mm), deep suction (easy passage)	pH 7.04, pCO₂ 78; CXR: NRDS; lung pulse on US
18 min	22:47	Chest compressions initiated; IV epinephrine (1:10,000) q3 min; PPV via resuscitation bag started concurrently	HR 50–60, SpO₂ 10%–20%
18 min – ∼1 h	22:47 – ∼23:29	All resuscitation measures (chest compressions, q3 min epinephrine, PPV via bag) continued without interruption (total ∼42 min)	Persistent bradycardia, hypoxia
∼1 h	∼23:29	Fragments expelled by chest compressions + PPV, cleared by ETT exchange; ROSC achieved	HR ↑ to 140, SpO₂ ↑ to 93%; lung pulse resolved; chest compressions discontinued
1 h 8 min	23:37	Admitted to NICU	Comatose, fixed pupils 4 mm, hypertonia
3.5 h	∼02:00 (Oct 25)	Seizure (focal clonic); phenobarbital	Bloody ETT secretions → suspected pulmonary hemorrhage
8 h	∼06:30 (Oct 25)	Laboratory critical values	WBC 0.43, neutrophils 0.05, platelets 98, coagulopathy
18 h	∼16:30 (Oct 25)	Head ultrasound	Intracranial hemorrhage confirmed
36 h	10:58 (Oct 26)	Family withdraws life support	Coma, no spontaneous breathing, multi-organ failure
36 h 19 min	14:17 (Oct 26)	Death declared	HR 0, ECG flatline
48–72 h	Oct 27–28	Blood culture & tracheal aspirate histopathology	Blood: ESBL-E. coli; Tracheal aspirate: decidual tissue

## 4 Conclusion

This fatal case demonstrates unexpected distal airway obstruction in a preterm infant caused by aspirated decidual tissue. Analysis of this case together with three previous reports (3–5) leads to three key points. First, this diagnosis should be suspected in any newborn with refractory apnea or inadequate ventilation after a complicated delivery, especially breech presentation. Second, standard algorithms (MRSOPA/DOPE) are insufficient for such solid airway obstruction; moreover, easy passage of a suction catheter—the “False-Patency” sign—can dangerously mislead clinicians rather than confirm airway patency. Third, despite the importance of rapid obstruction relief, early recognition is often delayed by the subtle presentation and limitations of current equipment. Consequently, addressing these catastrophic events requires two complementary actions: sharpening clinical awareness of the “False-Patency” sign, and promoting the long-term development of specialized, miniaturized airway visualization tools for preterm infants.

## Data Availability

The original contributions presented in the study are included in the article/Supplementary Material, further inquiries can be directed to the corresponding authors.
